# Does the Babinski sign predict functional outcome in acute ischemic stroke?

**DOI:** 10.1002/brb3.1575

**Published:** 2020-02-27

**Authors:** Jian‐Feng Qu, Yang‐Kun Chen, Gen‐Pei Luo, Dong‐Hai Qiu, Yong‐Lin Liu, Huo‐Hua Zhong, Zhi‐Qiang Wu

**Affiliations:** ^1^ Department of Neurology Dongguan People’s Hospital (Affiliated Dongguan Hospital, South Medical University) Dongguan China; ^2^ Faculty of Neurology Guangdong Medical University Dongguan China

**Keywords:** Babinski sign, brain MRI, functional status, ischemic stroke

## Abstract

**Objective:**

The aim of this prospective cohort study was to determine the incidence and neuroimaging risk factors associated with Babinski sign following acute ischemic stroke, as well as its relationship with the functional outcome of patients.

**Methods:**

A total of 351 patients were enrolled in the study within 7 days of acute ischemic stroke. The Babinski sign along with other upper motor neuron signs were examined upon admission and between days 1 and 3 and days 5 and 7 after admission. Neuroimaging parameters included site and volume of infarction and white matter lesions. All patients were followed up at 3 months. Functional outcome was assessed with the Lawton Activities of Daily Living scale and modified Rankin Scale.

**Results:**

Babinski sign was observed in 115 of 351 (32.8%) patients in the acute ischemic stroke. These patients had higher National Institutes of Health Stroke Scale (NIHSS) scores at admission and higher rates of atrial fibrillation and cardioembolism; higher frequencies of frontal, temporal, and limbic lobes and basal ganglia infarcts; and larger infarct volume. Higher NIHSS score and basal ganglia infarct were significant predictors of the presence of Babinski sign. After adjusting for confounds, the presence of Babinski sign did not predict poor functional outcome.

**Conclusion:**

The incidence of Babinski sign was 32.8% in the acute ischemic stroke. Severe infarction and basal ganglia infarct were independent predictors of Babinski sign. Although Babinski sign is common in acute ischemic stroke patients, it does not predict poor functional outcome 3 months later.

## Graphical abstract

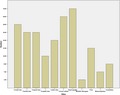

## 1 INTRODUCTION

Babinski sign, also known as the great toe sign, is the most sensitive and important indicator of an upper motor neuron lesion and was first described by Joseph Babinski in 1896. The sign consists of extension of the large toe and extension and fanning of the other toes during and immediately after the lateral plantar surface of the foot is stroked (Babinski, 1896). Several dozen surrogate responses have been described including Chaddock sign and Oppenheim sign based on alternative sites and types of stimulation, but all have the same physiological significance as the Babinski sign (Ghosh & Pradhan, 1998).

Ischemic stroke is a sudden interruption of blood supply to a structure due to occlusion or obstruction by a thrombus or embolus. The pyramidal tract is a large structure supplied by blood from many different arteries, with any occlusion leading to a wide variety of symptoms that include the presence of Babinski sign. However, this reflex has seldom been investigated in the acute ischemic stroke population by magnetic resonance imaging (MRI). A computed tomography study investigating the prevalence and reliability of upper motor neuron signs in the context of acute stroke reported a prevalence of Babinski sign of 64% (Louis, King, Sacco, & Mohr, 1995). Brain MRI can reveal structural details of acute ischemic stroke lesions and has greater sensitivity in detecting preexisting brain abnormalities.

It is not known whether the presence of Babinski sign is related to the functional outcome in stroke patients; however, if this is the case, it could provide prognostic information to neurologists and inform clinical decisions on how best to manage patients’ care in order to accelerate recovery. To address this issue, the present study investigated the incidence and neuroimaging risk factors of Babinski sign and the relevance to functional outcome in Chinese acute ischemic stroke patients.

## 2 MATERIALS AND METHODS

### 2.1 Study subjects and setting

**Figure 1:**
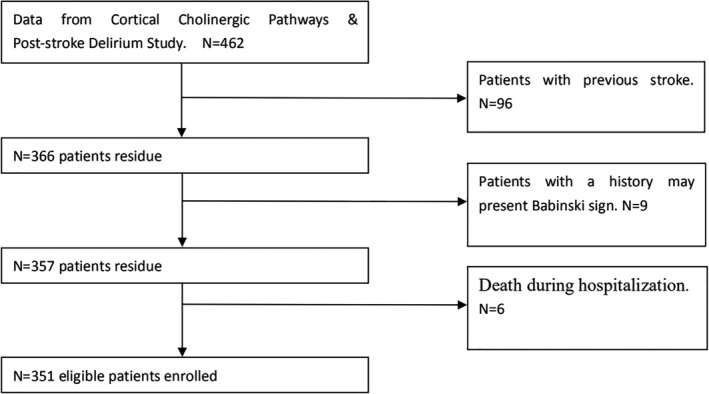
Patient selection process

This study was a retrospective analysis of prospectively collected data (Cortical Cholinergic Pathways and Post‐stroke Delirium Study) that was conducted at Division I, Department of Neurology, Dongguan People's Hospital between June 1, 2017, and June 1, 2018. The inclusion criteria for the study subjects were as follows: (a) age over 18 years; (b) first acute ischemic stroke occurring within 7 days before admission; and (c) underwent complete brain MRI examination. The exclusion criteria were as follows: (a) history of stroke; (b) transient ischemic attack, cerebral hemorrhage, subdural hematoma, or subarachnoid hemorrhage; (c) medical history that could affect Babinski sign (e.g., motor neurone disease, Parkinson's disease, cervical or thoracic spondylotic myelopathy, multiple sclerosis, or neuromyelitis optica spectrum disorders); (d) lack of complete clinical and brain MRI data (e.g., incomplete MRI sequences or poor‐quality images); (e) death during hospitalization; (f) refusal to provide written, informed consent by patients or their relatives; and (g) severe comorbidities such as organ dysfunction or malignancy. A total of 351 patients were eligible to participate in the study (Figure 1). The study protocol was approved by the Ethics Committee of Dongguan People's Hospital, and written informed consent was obtained from all participants. The study was registered at the Chinese Clinical Trial Registry (ChiCTR1800014982, URL: http://www.chictr.org.cn/index.aspx).

### 2.2 Collection of demographic and clinical data

Demographic and clinical information including age, sex, and vascular risk factors was prospectively recorded on a standard data collection form. The severity of stroke was assessed with the National Institutes of Health Stroke Scale (NIHSS) (Brott et al., 1989). The subtype of ischemic stroke was determined by neurologists during patient hospitalization based on the Trial of ORG 10172 in the Acute Stroke Treatment classification system (Adams et al., 1993).

### 2.3 Assessment of Babinski sign

Each patient was examined by one senior neurologist (JFQ, YLL, GPL, DHQ, or ZQW) according to a standard protocol following admission to the Stroke Unit. In addition to the Babinski sign, other upper motor neuron signs including Chaddock sign, Oppenheim sign, deep tendon hyperreflexia, and ankle clonus were recorded. The Babinski sign is not always observed within a few hours of acute ischemic stroke, but could be positive after admission (Louis et al., 1995). We tested for the Babinski sign and other upper motor neuron signs three times to confirm our results. The first examination was conducted at admission, with follow‐up examinations between days 1 and 3 and between days 5 and 7 after admission. These categorical variables were recorded as present or absent; the signs were considered as present if one of the three examinations yielded a positive result. If a patient's Babinski sign was negative at admission but was positive thereafter, it was defined as an altered Babinski sign; a positive sign from the time of admission was classified as persistent.

### 2.4 Follow‐up of participants

All participants were followed up for 3 months via telephone. Clinical outcome was determined according to the modified Rankin Scale (mRS), and functional status was evaluated with the Lawton Activities of Daily Living (ADL) scale (Lawton & Brody, 1969) comprising the basic and instrumental ADL. The former measures the capacity for toilet activity, feeding, dressing, grooming, ambulation, and bathing; the latter examines the patient's ability to use a telephone, shop, prepare food, perform housekeeping and laundry, use a mode of transportation, take responsibility for his/her own medications, and handle finances. The total ADL score was calculated as the sum of points for each item and ranged from 14 to 56, with a higher score reflecting worse performance. Poor functional outcome was defined as an ADL score higher than the 75% quartile, and a low mRS score was defined as ≥3 points. Recurrence of stroke and death during the follow‐up period was also recorded.

### 2.5 MRI assessment

Brain MRI scans including T1‐ and T2‐weighted imaging and diffusion‐weighted imaging (DWI) were performed on each subject with a Sonata 3.0‐T system (Siemens Medical) within 7 days of hospital admission. A DWI spin‐echo (SE) echo‐planar sequence (EPI; repetition time [TR]/echo time [TE]/excitation = 2,162/76/1, matrix = 128 × 128, field of view [FOV] = 230 mm, slice thickness/gap = 6/1 mm, EPI factor = 47, acquisition time = 25.9 s) with three orthogonally applied gradients and *b* values of 0 and 1,000 was used. Axial SE T1 (TR/TE/excitation = 488/15/1, FOV = 230 mm, slice thickness/gap = 6/1 mm, matrix = 256 × 256, and time of acquisition = 1 min 24.8 s) and turbo spin‐echo T2 (TR/TE/excitation = 3,992/110/2, turbo factor = 15, FOV = 230 mm, slice thickness/gap = 6/1 mm, matrix = 512 × 512, and acquisition time = 1 min 55.8 s) images were also acquired.

A neurologist (YKC) who was blinded to patients’ clinical information and assessment results evaluated the following MRI parameters.

#### 2.5.1 Brain infarct

The site and volume of acute lesions were determined from the DWI sequence. The sites of acute infarct were divided into cortical (frontal, temporal, parietal, occipital, and limbic lobes), subcortical (corona radiata and basal ganglia), and infratentorial (medulla oblongata, pons, mesencephalon, and cerebellum) regions. The total acute infarct area in DWI was measured from manually traced outlines. Acute infarct was defined as an area of restricted water diffusion with a *b* value of 1,000 combined with hypointensity on the corresponding apparent diffusion coefficient map. The total volume was calculated by multiplying the total area by the sum of the slice thickness and gap.

#### 2.5.2 White matter lesions (WMLs)

The severity of WMLs was graded on a 4‐point scale (Fazekas, Chawluk, Alavi, Hurtig, & Zimmerman, 1987). Periventricular and deep white matter hyperintensities were separately scored on fluid attenuation inversion recovery images.

Intrarater reliability tests were carried out by the same MRI rater (YKC) on 10 stroke patients. The intrarater agreement of MRI measurements was good to excellent (infarct volume intraclass coefficient, 0.83; site of infarction intrarater kappa, 0.85; and WML intrarater kappa, 0.82).

### 2.6 Statistical analysis

Statistical analyses were performed using SPSS v.24.0 for Windows (SPSS Inc.). Descriptive data are presented as a proportion, mean, or median as appropriate. Potential risk factors were compared between patients with and those without Babinski sign by univariate analysis. A backward elimination procedure was used in the logistic regression analysis; presence of Babinski sign was the dependent variable. Risk factors with a *p* value <.05 were analyzed by multivariate logistic regression analysis using a backward stepwise selection strategy. Correlation analysis was performed to test the collinearity between candidate independent variables. If the correlation coefficient between any two risk factors was ≥0.40, variables with a lower *p* value were entered into the logistic regression. We also analyzed the relationship between Babinski sign and low ADL and mRS scores after adjusting for potential confounds. The odds ratio (OR) of any independent risk factor was interpreted as the risk of poor outcome when all other risk factors were kept constant. The significance level was set at .05 (two‐sided).

## 3 RESULTS

**Figure 2:**
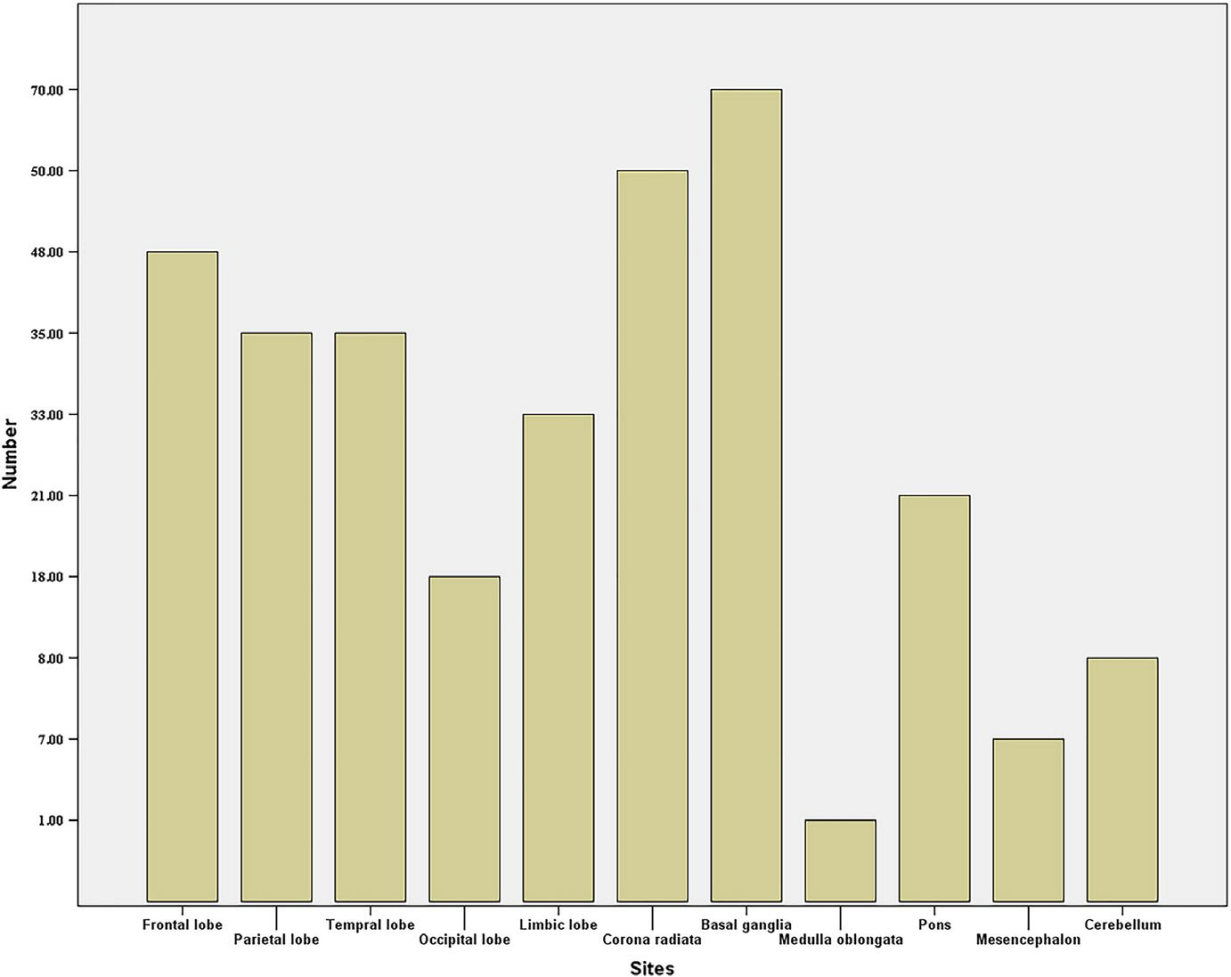
Distribution of patients with Babinski sign

Of the 351 eligible patients, 249 (70.9%) were male and 102 (29.1%) were female, with a mean age of 61.1 years (range, 19–91 years). The median NIHSS score at the time of admission was 4 (range, 0–28). The baseline characteristics of the study population are summarized in Table 1. The distribution of acute infarct according to Babinski sign is shown in Figure 2.

**Table 1:** Demographic and clinical characteristics of the study population

Characteristic	Mean (*SD*)/median (IQR)/*n* (%) (*n* = 351)
Age (years)^a^	61.1 (14.6)
Males^b^	249 (70.9%)
Hypertension^b^	238 (67.8%)
Diabetes mellitus^b^	87 (24.8%)
Atrial fibrillation^b^	40 (11.4%)
NIHSS on admission^c^	4 (2–7)
Intravenous thrombolysis^b^	20 (5.7%)
Stroke subtype^b^	
Large artery	149 (42.5%)
Small artery	101 (28.8%)
Cardioembolism	35 (10%)
Other etiologies	15 (4.3%)
Unknown etiology	51 (14.5)
Location of infarct^b^	
Frontal lobe	112 (31.9%)
Parietal lobe	88 (25.1%)
Temporal lobe	74 (21.1%)
Occipital lobe	49 (14%)
Limbic lobe	66 (18.8%)
Corona radiata	156 (44.4%)
Basal ganglia	159 (45.3%)
Medulla oblongata	8 (2.3%)
Pons	57 (16.2%)
Mesencephalon	13 (3.7%)
Cerebellum	30 (8.5%)
Cortical region	151 (43%)
Subcortical region	245 (69.8%)
Infratentorial region	89 (25.4%)
Infarct volume^c^	1.95 (0.755–9.275)
PVH^c^	1 (0–2)
DWMH^c^	1 (0–1)
Babinski sign (+)^b^	115 (32.8%)
Chaddock sign (+)^b^	53 (15.1%)
Oppenheim sign (+)^b^	56 (16%)
Hyperreflexic sign (+)^b^	31 (8.8%)
Clonus sign (+)^b^	25 (7.1%)

Abbreviations: DWMH, deep white matter hyperintensities; IQR, interquartile range; NIHSS, National Institutes of Health Stroke Scale; PVH, periventricular hyperintensities.

a Mean (*SD*).

b n (%).

c Median (IQR).

### 3.1 Incidence of Babinski sign

Babinski sign was observed in 115 of 351 (32.8%) patients in the acute phase of ischemic stroke. Of the 115 patients, 17 (14.8%) were negative for the Babinski sign at admission but switched to a positive status thereafter. The other manifestations included Chaddock sign (*n* = 53), Oppenheim sign (*n* = 56), deep tendon hyperreflexia (*n* = 31), and ankle clonus (*n* = 25).

### 3.2 Univariate analysis

In the univariate analysis, patients presenting with Babinski sign had higher NIHSS scores at admission and more frequent atrial fibrillation and cardioembolism than their counterparts without Babinski sign. They also had higher rates of infarct in the frontal, temporal, and limbic lobes and basal ganglia as well as larger infarct volume (Table 2).

**Table 2:** Risk factors for Babinski sign in the univariate analysis

Variable	Subjects with Babinski sign (*n* = 115)	Subjects without Babinski sign (*n* = 236)	*t*/*χ* ^2^/*z* value	*p* value
Age (years)^a^	61.7 (15.4)	60.8 (14.2)	1.788	.182
Males^b^	80 (69.6%)	169 (71.6%)	0.157	.692
Hypertension^b^	70 (60.9%)	168 (71.2%)	3.77	.052
Diabetes mellitus^b^	29 (25.2%)	58 (24.6%)	0.017	.896
Atrial fibrillation^b^	22 (19.1%)	18 (7.6%)	10.133	.001
NIHSS on admission^c^	7 (3–12)	3 (1–5)	−7.074	< .001
Intravenous thrombolysis^b^	8 (7%)	12 (5.1%)	0.504	.478
Stroke subtype^b^			11.2	.024
Large artery	47 (40.9%)	102 (43.2%)		
Small artery	30 (26.1%)	71 (30.1%)		
Cardioembolism	20 (17.4%)	15 (6.4%)		
Other etiologies	5 (4.3%)	10 (4.2%)		
Unknown etiology	13 (11.3%)	38 (16.1%)		
Location of infarct^b^				
Frontal lobe	48 (41.7%)	64 (27.1%)	7.607	.006
Parietal lobe	35 (30.4%)	53 (22.5%)	2.619	.106
Temporal lobe	35 (30.4%)	39 (16.5%)	8.991	.003
Occipital lobe	18 (15.7%)	31 (13.1%)	0.408	.523
Limbic lobe	33 (28.7%)	33 (14%)	10.962	.001
Corona radiata	50 (32.1%)	106 (44.9%)	0.065	.799
Basal ganglia	70 (60.9%)	89 (37.7%)	16.734	< .001
Medulla oblongata	1 (0.9%)	7 (3%)	1.526	.217
Pons	21 (18.3%)	36 (15.3%)	0.514	.473
Mesencephalon	7 (6.1%)	6 (2.5%)	2.724	.099
Cerebellum	8 (7%)	22 (9.3%)	0.554	.457
Cortical region	58 (50.4%)	93 (39.4%)	3.836	.5
Subcortical region	87 (75.5%)	158 (66.9%)	2.778	.096
Infratentorial region	28 (24.3%)	61 (25.8%)	0.092	.762
Infarct volume^c^	4.365 (1–20.79)	1.395 (0.647–6.207)	−4.233	< .001
PVH^c^	1 (0–2)	1 (0–2)	−0.565	.572
DWMH^c^	1 (0–1)	1 (0–1)	−0.541	.589

Abbreviations: DWMH, deep white matter hyperintensities; NIHSS, National Institutes of Health Stroke Scale; PVH, periventricular hyperintensities.

a Mean (*SD*), *t* test.

b *n*(%), chi‐square test.

c *M* (upper quartile–lower quartile), Mann‐Whitney *U* test.

### 3.3 Multivariate logistic regression analysis

Infarct volume was not included in the multivariate logistic regression model as it was highly correlated with NIHSS score (r = .543). Atrial fibrillation, NIHSS score at admission, stroke subtype, and infarct in the frontal, temporal, and limbic lobes and basal ganglia were entered into the model. A higher NIHSS score and basal ganglia infarct were significant predictors of Babinski sign. The presence of a basal ganglia infarct had an OR of 1.738 (95%CI = 1.055–2.863, *p* = .03) (Table 3). Additionally, there was no difference in the rates of poor functional outcome between patients with persistent versus altered Babinski sign (poor ADL, 37% vs. 23.5%, *p* = .134; poor mRS, 60.5% vs. 47.1%, *p* = .306, respectively). After adjusting for confounds, the presence of Babinski sign (including altered Babinski sign) was not a predictor of poor outcome, as measured by the ADL scale and mRS (Table 4).

**Table 3:** Multivariate logistic regression analysis of risk factors for subjects with Babinski sign

Variable	*β*	OR (95% CI)	*p* value
Atrial fibrillation	0.402	1.495 (0.681–3.283)	.316
NIHSS on admission	0.149	1.161 (1.102–1.222)	<.001
Stroke subtype	0.012	1.012 (0.852–1.203)	.889
Frontal lobe	0.367	1.443 (0.853–2.44)	.172
Temporal lobe	−0.365	0.694 (0.337–1.43)	.322
Limbic lobe	0.175	1.191 (0.561–2.53)	.648
Basal ganglia	0.553	1.738 (1.055–2.863)	.03

Abbreviation: NIHSS, National Institutes of Health Stroke Scale.

**Table 4:** Outcome of subjects with Babinski sign^a^

Variable	mRS			ADL		
	*β*	OR (95% CI)	*p* value	*β*	OR (95% CI)	*p* value
Babinski sign (+)	−0.11	0.989 (0.509–1.922)	.957	0.194	1.214 (0.598–2.465)	.592
Altered Babinski sign	0.106	1.112 (0.278–4.44)	.881	0.431	1.539 (0.422–5.617)	.514

a Adjusted for age, NIHSS score on admission, stroke subtype, and atrial fibrillation.

## 4 DISCUSSION

In this present study, we found that the incidence of Babinski sign was 32.8% in the acute stage of overall ischemic stroke. The presence of Babinski sign was associated with higher NIHSS score and infarct in the basal ganglia. However, patients with Babinski sign did not have a worse functional outcome than those without this reflex, suggesting that the presence or absence of Babinski sign cannot be used to predict the prognosis of acute ischemic stroke patients.

Babinski sign was more frequently observed in our patients than other upper neuron signs of acute ischemic stroke, with an incidence of 32.8%. A retrospective review of 1,600 cases of pyramidal tract syndrome revealed that only 30% of cases were considered acute (Lassek, 1945). An investigation of acute hemorrhagic and ischemic stroke patients found an incidence of Babinski sign of 64.8%. The divergent findings of these studies may be attributable to the heterogeneous cohorts and variable periods of examination (Louis et al., 1995). In our study, patients were evaluated within 1 week after hospital admission for acute ischemic stroke, yielding a more homogeneous study population and defined examination period. Thus, Babinski sign has little clinical relevance for the management of acute ischemic stroke.

Our results showed that higher NIHSS score at admission predicted the presence of Babinski sign. The NIHSS is widely used to assess stroke severity (Lyden, 2017) and mainly reflects the motor ability of proximal limbs; it does not include a detailed assessment of the cranial nerves, and relatively low scores may be observed in patients with disabling infarctions of the brainstem or cerebellum (Kasner, 2006). A higher NIHSS score is correlated with more severe motor lesions, indicating a higher probability of a positive Babinski sign.

Few studies have investigated the relationship between lesion pattern and Babinski sign by brain MRI. It was previously suggested that cortical but not subcortical lesions predict the Babinski sign (Deng, Jia, Zhang, Guo, & Yang, 2013), the presence of which always implies upper motor neuron dysfunction in the brain or spinal cord in adults (Ambesh, Paliwal, Shetty, & Kamholz, 2017). The pyramidal tract is concentrated in the basal ganglia after emerging from the cortex (Ashby, Andrews, Knowles, & Lance, 1972), and infarcts involving this structure can increase the risk of Babinski sign. Indeed, our study showed that basal ganglia infarcts were a predictor of this reflex.

The Babinski sign is a very important part of neurological examinations as it is the most important indicator of an upper motor neuron lesion (Babinski, 1896). Our results indicate that the presence of Babinski sign does not predict poor functional outcome of acute stroke patients at the 3‐month follow‐up. The mRS reflects the overall degree of disability of patients including physical function and prognosis (van Swieten, Koudstaal, Visser, Schouten, & Gijn, 1988), whereas the ADL scale primarily evaluates memory and executive functions (Fukui & Lee, 2009). The Babinski sign is a common manifestation in acute ischemic stroke patients that is generally attributed to a pyramidal tract lesion and is unrelated to stroke severity (Lance, 2002). However, the aim of our study was to determine whether it could predict functional outcome in stroke, rather than to question its importance.

Our study had the following advantages: (a) We examined consecutively recruited acute ischemic stroke patients; (b) we evaluated comprehensive neuroimaging parameters and obtained detailed data on location of infarcts and presence of WMLs; and (c) we analyzed the relationship between Babinski sign and clinical outcome. However, there were also some limitations to the study. Firstly, the sample size was relatively small; secondly, we did not evaluate other upper neuron signs such as spasticity and the Gordon sign. Finally, we did not assess upper neuron signs in the follow‐up and were therefore unable to adequately evaluate stroke severity.

In conclusion, a higher NIHSS score and basal ganglia infarct predict the presence of Babinski sign. However, Babinski sign is unrelated to functional outcome of acute stroke patients at the 3‐month follow‐up. Thus, neurologists should pay more attention to the functional status in stroke patients based on manifestations other than Babinski sign.

## CONFLICT OF INTEREST

The authors declare that they have no competing interest.

## AUTHOR CONTRIBUTIONS

JF and YK conceived and designed the research. JF, GP, DH,YL, and HH collected the data. JF, YK, and HH analyzed the data and prepared the tables. ZQ revised the manuscript critically for important intellectual content.

## Data Availability

Requests for access to the data and analysis tools in this article will be openly considered. The data that support the findings of this study are available from the corresponding author upon reasonable request.
